# A Decade of Dedication: Pioneering Perspectives on Neurological Diseases and Mental Illnesses

**DOI:** 10.3390/biomedicines12051083

**Published:** 2024-05-13

**Authors:** Masaru Tanaka, László Vécsei

**Affiliations:** 1 HUN-REN-SZTE Neuroscience Research Group, Hungarian Research Network, University of Szeged, Danube Neuroscience Research Laboratory, Tisza Lajos krt. 113, H-6725 Szeged, Hungary; vecsei.laszlo@med.u-szeged.hu; 2 Department of Neurology, Albert Szent-Györgyi Medical School, University of Szeged, Semmelweis u. 6, H-6725 Szeged, Hungary

## 1. Introduction

Welcome to *Biomedicines*’ 10th Anniversary Special Issue, a journey through the human mind’s labyrinth and complex neurological pathways. This edition, focused on “Translational Laboratory and Experimental Medicine for Neurological Diseases and Mental Illnesses”, presents 21 pioneering papers that explore the enigmas of the brain and its remarkable ability to heal and adjust. We investigate the various impacts of time on neural circuits and cognitive responses. Our research spans studying how the brain can adjust and recuperate following a stroke, a process known as neuroplasticity, to exploring the intricate link between age and behavior [1,2,3,4,5,6]. 

We are particularly interested in the neural mechanisms that underpin these mechanisms, such as the role of neural circuits and their plasticity in cognitive responses [7,8,9,10,11]. By investigating neural activity and connectivity, we hope to gain insight into brain adaptation [12,13,14]. This entails investigating how these changes affect cognitive functions such as memory and decision-making, as well as their implications for cognitive development and disorders [15,16,17,18,19,20,21]. Hence, we may be able to uncover the complex mechanisms that underpin neurodegenerative disorders and investigate potential therapeutic strategies that hold promise for novel treatments [22,23,24,25,26,27,28]. 

Preclinical research plays a crucial role in understanding neuropsychiatric conditions [29,30,31,32]. By conducting studies in vitro and in vivo, researchers gather valuable data that would be impractical to obtain directly from humans [33,34,35,36,37,38]. These preclinical findings, combined with ongoing clinical studies, help us better understand the behavioral aspects of neuropsychiatric disorders [39,40,41]. Computational and inferential methods also contribute to new approaches to treating neurological and psychiatric disorders by helping to unravel the underlying pathology [42,43,44,45,46,47,48]. Integrating interdisciplinary methods further optimizes drug development research, leading to the evaluation of potential lead compounds [27,49,50,51,52]. Promising interventions, such as brain stimulation, have the potential to transform treatment and pave the way for new and more effective drugs for neurological and psychiatric conditions [53,54,55,56,57].

In our quest to break barriers and unveil unknowns, we also delve into the realm of mental health, exploring the biochemical basis of suicidal thoughts and the relationship between mental illness and pain. Whether you are a clinician, researcher, or simply curious about the complexities of the human mind, this collection of articles promises to challenge conventional wisdom and expand your horizons [58,59,60]. Join us in commemorating ten years of groundbreaking exploration and advancement in the realm of biomedicine.

## 2. Special Issue Articles

### 2.1. Stroke and Neuroplasticity: Unraveling the Brain’s Resilience

The complex interaction between stroke and neuroplasticity is central to the process of post-stroke recovery [61,62,63]. When a stroke happens, it disrupts the complex neural pathways, leading to a sequence of impairments [64,65,66]. Nonetheless, the brain, as the master of adaptation, makes use of its hidden asset: neuroplasticity. This remarkable phenomenon facilitates neuroplasticity, which involves the brain’s ability to reorganize itself by forming new synaptic connections and redirecting functions to unaffected areas [67,68,69]. The present section explores five fascinating studies that shed light on the interaction between stroke consequences and the brain’s extraordinary capacity for recovery. The authors of the referenced articles investigate brain legion prediction via dizziness, cognitive symptoms caused by subcortical damage, the gut microbiota in stroke patients, the effect of alcohol on neurogenesis, and the potential of virtual reality in cognitive rehabilitation. These articles provide a promising and resilient perspective on overcoming neurological challenges (Table 1).

Vertigo is a rare symptom in people who have recently suffered from a stroke, and there is currently a limited understanding of its significance [91]. d’Annunzio et al. make a notable contribution by showing that vertigo in patients with acute stroke can be used as an indicator of the stroke’s location, specifically in the cerebellum and/or brainstem [70]. However, it does not have an impact on early outcomes or increase the risk of mortality during hospitalization. Cortical damage is commonly associated with cognitive dysfunction, while subcortical damage is an aspect of cognitive dysfunction that is frequently overlooked in research [92]. Shimmyo and Obayashi improve our understanding of cognitive deterioration following pontine stroke, a frequently overlooked condition due to the incorrect belief that subcortical damage is less likely to induce cognitive dysfunction [71]. The study employs two neuroimaging techniques to better understand the neurophysiology that underpins cognitive decline. The study results suggest that the degree of cognitive decline may be related to the responses observed in the supplementary motor area. This phenomenon may be attributed to the breakdown of hierarchical cognitive processing in the fronto–ponto–cerebellar–thalamic loop.

A growing body of evidence suggests that disorders of the central nervous system (CNS) can be linked to peripheral body regions [93,94]. Park et al. investigated the gut microbiota in individuals who have suffered from strokes, uncovering significant imbalances in both the taxonomic composition and functional characteristics of the microbiota when compared to a group of healthy individuals. Patients who have experienced a stroke exhibit changes in their gut microbiota, which may be a sign of malnutrition. Adjusting their diet could help restore a healthy balance of gut bacteria, leading to better outcomes and a decrease in disability and death rates in stroke patients.

Alcohol consumption is well known to affect the risk and prognosis of ischemic stroke [95,96]. Li et al. examined the effects of light alcohol consumption (LAC) on the growth of new neurons in the brain in the context of ischemic stroke [73]. The findings of their study indicate that LAC can considerably enhance neurogenesis in both normal conditions and after an ischemic stroke. This process has the potential to minimize brain damage and enhance locomotor activity, suggesting that LAC may have a protective effect against ischemic stroke. There is an increasing need for more objective outcome measures in cognitive rehabilitation (CR) for stroke patients [97,98,99]. Gangemi et al. contribute to the field of CR by showing that a virtual reality-based approach has the potential to effectively promote neuroplastic changes in patients with chronic ischemic stroke [74]. This is supported by significant improvements in electroencephalogram (EEG)-related neural activity and variations in power spectral density in the alpha and beta band powers (Table 1).

### 2.2. Age and Behavioral Studies: Unraveling the Complexities of Lifespan Influence

We gain more insight into the complex relationship between age and behavior as we investigate the various ways that aging affects brain networks and cognitive processes [100,101,102]. This section launches a journey through five illuminating studies, each shedding light on the dynamic relationship between age and behavioral outcomes. The section showcases various aspects of scientific research, including the vulnerability of mice to ketamine, the complex relationship between the dosage and effects of ketamine, the connection between melatonin and anxiety in C57/B6J mice, the role of platelet mitochondria, and the involvement of noradrenaline in regulating learned and innate behaviors in rats lacking the dopamine transporter. These articles invite us to reflect on the complex interplay of age, behavior, and the constantly changing brain.

Ketamine is frequently abused as a psychedelic substance [103,104,105]. Chen et al. examined the impact of ketamine on glutamatergic neurotransmission, which plays a vital role in memory retention, addiction, and psychosis [75]. The authors of the study investigate the varying sensitivity to ketamine in mice of different ages and strains. The results indicate that an individual’s response to ketamine, as observed through their locomotor behavior, is determined by biological factors and can differ depending on dosage and age. 

The production of melatonin decreases as one ages, and its effectiveness may vary depending on age [106,107,108]. The study conducted by Nasini et al. examines the impact of melatonin on anxiety-related behavior and the circuit connecting the medial prefrontal cortex and dorsal hippocampus in both adolescent and adult mice [76]. The results emphasize the variations in the effects of melatonin based on age, indicating that age can have a substantial influence on outcomes. 

Mitochondrial dysfunction, characterized by a decline in mitochondrial respiratory function and an increase in reactive oxygen species production, is a key cellular hallmark of aging and neurodegenerative diseases [52,109,110]. Fišar et al. utilized platelets as a model to assess age-related mitochondrial parameters and the influence of cognitive impairment on these parameters [77]. The study shows age-dependent changes in mitochondrial function in platelets but no significant difference between individuals with and without cognitive impairment. Platelet mitochondrial respiration may serve as a promising biomarker for aging and a target for interventions aimed at combating aging and neurodegenerative processes.

Developing focused treatment approaches for attention-deficit hyperactivity disorder requires investigation of the underlying mechanisms involving dopamine dysregulation [111,112,113]. Volnova et al. examined the impacts of guanfacine, an α2A-adrenoceptor agonist, on the behavior and brain activity of dopamine transporter knockout rats [78]. Guanfacine has been shown to improve spatial working memory and pre-pulse inhibition in dopamine transporter knockout rats. This supports the role of noradrenergic modulation in attention regulation and suggests potential combined treatments to maintain dopamine–norepinephrine balance (Table 1).

### 2.3. Neuropsychiatric Disorders and Treatments: Unraveling Pathways and Novel Approaches

Neurodegenerative disorders pose a substantial and increasing public health issue, impacting a considerable population worldwide [114,115,116]. These conditions, including Alzheimer’s disease, Parkinson’s disease (PD), multiple sclerosis (MS), and amyotrophic lateral sclerosis (ALS), are defined by the gradual deterioration and depletion of nerve cells in the brain and spinal cord [117,118,119,120]. This section is focused on recent advances in understanding these diseases, their causes, and potential treatments including neuroprotection, highlighting innovative research that offers hope for new therapies [121]. The six articles we feature cover a broad spectrum of neurodegeneration, each focusing on a different aspect of these complex conditions.

The enteric nervous system (ENS) is intricately linked to the CNS and plays an important role in the pathophysiology of PD [122,123,124]. Montanari et al. discuss the early involvement of the ENS in PD pathogenesis, with α-synuclein (α-syn) aggregation occurring before CNS symptoms [79]. By proposing the ENS as a target for potential new PD therapies, this could provide insights into brain health and advance the development of novel therapeutic options.

Little is known about the interactions between ubiquitin-like 3 (UBL3) and alpha-synuclein (α-syn), and their modulation by drugs, which are relevant for understanding and treating α-synucleinopathies [125,126,127]. Chen et al. examined the interaction between UBL3 and α-syn in order to comprehend its function in α-synucleinopathies [80]. UBL3 interacts with α-syn, and this interaction is modulated by osimertinib, an inhibitor of the epidermal growth factor receptor pathway. This study advances the field by identifying the UBL3 pathway as a potential new therapeutic target for α-synucleinopathies.

Neuroinflammation is increasingly recognized as a significant factor in a variety of brain diseases, with microglia and monocytes playing an important role in the robust activation of the NOD-, LRR- and pyrin domain-containing protein 3 (NLRP3) inflammasome [128,129,130]. Chiarini et al. discuss the regulation of NLRP3 and its involvement in diverse neurological disorders [81]. The authors acknowledge the absence of proof regarding the impact of NLRP3 inhibition on human diseases and emphasize the possibility of other inflammasomes stepping in to fill the gap. They advocate for the use of human neural cell-based models to gain a deeper understanding of these diseases and develop more effective treatment strategies.

Anomalies in the tryptophan (Trp)-kynurenine (KYN) metabolic system have been detected in individuals with MS [131,132,133]. However, the specific profile of KYN metabolites in progressive MS is still uncertain. Polyák et al. examined KYN metabolite levels in a cuprizone-induced mouse model of demyelination [82]. The authors show significant reductions in specific KYN metabolites, suggesting that these metabolites are potential biomarkers for personalized MS treatment.

Sodium voltage-gated channel alpha subunit 1 (SCN1A) gene mutations cause cellular immaturity in neurons, resulting in delayed maturation and reduced excitability, both of which contribute to the development of febrile seizures [134,135,136]. Scalise et al. investigated the effect of SCN1A gene mutations from a well-characterized Italian family on neurons derived from induced pluripotent stem cell-derived neurons [83]. The mutations cause reduced excitability in neurons as well as intrinsic cellular immaturity. The authors provide strong evidence that SCN1A gene mutations play a role in the development of febrile seizures, highlighting the potential of diseased neurons for personalized therapy and ex vivo drug screening for human epileptic disorders.

The secretome of dental pulp stem cells (DPSCs) on motoneurons in ALS demonstrated neuroprotective effects; however, the mechanism of action remains unknown [137,138,139]. Younes et al. investigated the effects of the DPSC secretome on the survival, axonal length, and electrical activity of cultured wild-type and superoxide dismutase 1 (SOD1) G93A motoneurons, as well as the roles of two DPSC-secreted factors, growth/differentiation factor 15 (GDF15) and heparin-binding epidermal growth factor-like growth factor (HB-EGF) [84]. The secretome of DPSCs has neuroprotective effects on motoneurons and could be a therapeutic candidate for ALS, highlighting the roles of GDF15 and HB-EGF, two DPSC-secreted factors that protect motor neurons from nitric oxide-induced death.

Individuals with spinal cord injury (SCI) experience rapid and debilitating muscle and bone loss, necessitating the development of effective bone mass preservation and maintenance strategies to reduce the risk of fragility and fracture in these vulnerable populations [85,140,141,142]. Leone et al. investigated the pathophysiology and risk factors of muscle and bone loss after SCI, the mechanisms that contribute to this loss, and current and future pharmacological and non-pharmacological therapies for reducing or eliminating neurogenic bone loss after SCI [85]. Pharmacological and non-pharmacological treatments can lessen or completely prevent neurogenic bone loss following SCI. Additionally, people with SCI have more rapid and severe bone and muscle loss because of a number of different factors (Table 1).

### 2.4. Mental Health and Disorders: Breaking Barriers and Unveiling Secrets

Mental health is a fundamental component of our general state of being, impacting our cognitive processes, emotions, and social interactions [143,144,145]. This section explores a wide range of research articles that provide insights into different aspects of mental health and disorders. These studies provide valuable insights into the relationship between mental illness and pain, as well as the biochemical basis of suicidal thoughts. Let us delve into the complex neural pathways, biological indicators, and psychological phenomena that influence our comprehension of mental health [146]. 

Despite the importance of intentional forgetting (IF) in daily performance, psychological well-being, and memory functioning, the neuropsychological mechanisms underlying successful IF are unknown [147,148,149]. Gamboa et al. investigated the neural correlates of IF using two meta-analytic algorithms, activation likelihood estimation, and latent Dirichlet allocation, and evaluated the proposed neurobiological models’ compatibility with existing brain imaging data [86]. IF involves the interaction of two networks: a primarily right-lateralized frontal–parietal circuit and a less constrained supportive network that includes frontal–hippocampal interactions. In support of the inhibitory or thought suppression hypothesis, the study also discovered a neural signature of IF that is consistent across various experimental paradigms and may open new avenues for developing effective clinical interventions.

Gaze cueing plays an important role in the reflexive orientation of attention and its susceptibility to context [149,150,151]. However, the distinct functional roles of the amygdala and the superior temporal lobe, particularly the superior temporal sulcus (STS), in gaze processing remain unknown, as does the interaction of contextual factors with gaze-cueing [152,153,154]. Battaglia et al. investigated the neural bases of gaze cueing and gaze direction perception, how contextual factors interact with the gaze shift of attention, and the distinct functional roles of the amygdala and STS in gaze perception [87]. The amygdala and the STS are important components in gaze perception, and gaze-cueing is influenced by a variety of context-specific factors. The idea of invariant representation is a useful framework for further research, highlighting the disparities in attempts to characterize the distinct functional roles of these regions in the processing of gaze. Th authors emphasize the role of the amygdala and the STS in gaze perception and introduce the concept of invariant representation as a valuable conceptual framework for future research on the perceptual processing of gaze within the STS.

The differences in serum brain-derived neurotrophic factor (BDNF) levels during pharmacotherapy in major depressive disorder (MDD) patients, particularly between treatment-response and treatment-nonresponse groups, remain unclear [155,156,157]. Yoshimura et al. studied changes in serum BDNF concentrations in first-episode, drug-naive MDD patients during antidepressant treatment and compared them to treatment-response and treatment-nonresponsive groups [88]. In first-episode, drug-naive MDD patients, serum BDNF levels did not differ significantly between treatment-response and treatment-nonresponse groups. However, the responder group showed statistically significant changes in serum BDNF, implying that the changes in serum BDNF may differ between the two groups and that measuring serum BDNF has the potential to be a useful predictor of pharmacotherapy in these patients. The authors demonstrate that serum BDNF measurement has the potential to be a useful predictor of pharmacotherapy in first-episode, drug-naive MDD patients.

Despite the emphasis on neurobiological underpinnings and the poor predictive accuracy of many sociodemographic risk factors and prognostic markers, understanding and predicting suicide remain significant challenges [158,159,160]. Cremone et al. examined the relationship between blood levels of serotonin, BDNF, Trp and its metabolites, interleukin-6 (IL-6), and homocysteine levels and suicidality in adults with autism and explored how these biochemical parameters may be linked to an elevated risk of suicide [89]. There is a link between suicidality and autism, and suicidality is associated with elevated homocysteine and IL-6 levels, as well as decreased Trp and KYNA levels. The authors show a possible transnosographic link between these biochemical parameters and increased suicide risk, which potentially improves our understanding and prediction of suicide.

Despite the known association between psychological events and pain intensity, there is no comprehensive mathematical model that accurately captures the multidimensional nature of pain, particularly low back pain, and its relationship with psychological factors [161,162,163]. Parolini et al. investigated the development of a mathematical representation of the International Association for the Study of Pain (IASP) pain model, using an artificial neural network to identify patterns in the relationship between various variables related to low back pain, as well as how these patterns differ between groups with altered patterns in the context of low back pain [90]. The authors show a direct correlation between psychological and pain events in the context of low back pain, suggesting that mental illness can exacerbate pain episodes and impact functionality. They also found that the developed artificial neural network model was able to identify patterns and relationships between variables and differentiate groups with altered patterns (Table 1).

## 3. Conclusions

This 10th Anniversary Special Issue of *Biomedicines* has thoroughly examined the field of translational laboratory and experimental medicine in relation to neurological diseases and mental illnesses. Within this compilation, scholars have extensively examined the intricate mechanisms that form the basis of these conditions, offering novel perspectives on possible therapeutic strategies and interventions. In addition to improving our understanding of the human mind, this Special Issue has facilitated groundbreaking advances in the diagnosis, treatment, and prevention of neurological and psychiatric disorders, such as the use of neuromodulation techniques. These techniques have shown promise in the treatment of various neurological and neuropsychiatric disorders, such as depression, anxiety, PD, and chronic pain [21,164,165,166,167]. The Special Issue on “Translational Laboratory and Experimental Medicine for Neurological Diseases and Mental Illnesses” is a testament to our unwavering dedication and innovation in the field of biomedicine as we celebrate a decade of pioneering exploration. The 21 papers presented herein demonstrate the diligent work of researchers and clinicians in understanding the intricacies of brain function and mental health. Their research provides encouraging perspectives on innovative therapeutic approaches and possible advancements. Our future goal is to connect the work carried out in laboratories with real-world applications, with the common objective of improving the lives of people affected by neurological disorders and mental illnesses.

## Figures and Tables

**Table 1:** Major subjects covered in the Special Issue “10th Anniversary of *Biomedicines*—Translational Laboratory and Experimental Medicine for the Sake of Neurological Diseases and Mental Illnesses”.

Subjects		Ref.
Stroke and neuroplasticity		
	Vertigo and stroke	[70]
	Pontine stroke effects	[71]
	Gut dysbiosis and stroke	[72]
	Alcohol and neurogenesis	[73]
	VR cognitive training	[74]
2. Age and behavioral studies		
	Ketamine vulnerability	[75]
	Melatonin and anxiety	[76]
	Platelet mitochondrial changes	[77]
	Guanfacine and behavior	[78]
3. Neuropsychiatric disorders and treatments		
	Enteric nervous system and PD	[79]
	UBL3 and alpha-synuclein	[80]
	NLRP3 inflammasome in brain diseases	[81]
	Metabolism and MS	[82]
	Stem cells and febrile seizures	[83]
	Stem cells and ALS	[84]
	Rehabilitation and spinal cord injury	[85]
4. Mental health and disorders		
	Intentional forgetting	[86]
	Gaze perception	[87]
	BDNF and major depression	[88]
	Autism and suicidal thoughts	[89]
	AI and mental illness	[90]

Abbreviations: AI: artificial intelligence, ALS: amyotrophic lateral sclerosis, BDNF: brain-derived neurotrophic factor, MS: multiple sclerosis, NLRP3: NOD-, LRR-, and pyrin domain-containing protein 3, PD: Parkinson’s disease, UBL3: ubiquitin-like 3, VR: virtual reality.

## Data Availability

Data sharing is not applicable to this article.

## Abbreviations

ALS	Amyotrophic lateral sclerosis
α-syn	Alpha-synuclein
BDNF	Brain-derived neurotrophic factor
CNS	Central nervous system
CR	Cognitive rehabilitation
DPSCs	Pulp stem cells
ENS	Enteric nervous system
GDF15	Growth/differentiation factor 15
HB-EGF	Heparin-binding epidermal growth factor-like growth factor
IF	Intentional forgetting
IL-6	Interleukin-6
KYN	Kynurenine
LAC	Light alcohol consumption
MDD	Major depressive disorder
MS	Multiple sclerosis
NLRP3	NOD-, LRR-, and pyrin domain-containing protein 3
PD	Parkinson’s disease
SCN1A	Sodium voltage-gated channel alpha subunit 1
SOD1	Superoxide dismutase 1
SCI	Spinal cord injury
STS	Superior temporal sulcus
Trp	Tryptophan
UBL3	Ubiquitin-like 3

## References

[1] Campos B., Choi H., DeMarco A.T., Seydell-Greenwald A., Hussain S.J., Joy M.T., Turkeltaub P.E., Zeiger W. (2023). Rethinking Remapping: Circuit Mechanisms of Recovery after Stroke. J. Neurosci..

[2] Gregorio F., Battaglia S. (2024). The intricate brain-body interaction in psychiatric and neurological diseases. Adv. Clin. Exp. Med..

[3] Brewster K.K., Golub J.S., Rutherford B.R. (2021). Neural circuits and behavioral pathways linking hearing loss to affective dysregulation in older adults. Nat. Aging.

[4] Battaglia S., Avenanti A., Vécsei L., Tanaka M. (2024). Neurodegeneration in Cognitive Impairment and Mood Disorders for Experimental, Clinical and Translational Neuropsychiatry. Biomedicines.

[5] Luo L. (2021). Architectures of neuronal circuits. Science.

[6] Battaglia S., Garofalo S., di Pellegrino G. (2018). Context-dependent extinction of threat memories: Influences of healthy aging. Sci. Rep..

[7] Andrade-Talavera Y., Fisahn A., Rodríguez-Moreno A. (2023). Timing to be precise? An overview of spike timing-dependent plasticity, brain rhythmicity, and glial cells interplay within neuronal circuits. Mol. Psychiatry.

[8] Tortora F., Hadipour A.L., Battaglia S., Falzone A., Avenanti A., Vicario C.M. (2023). The role of serotonin in fear learning and memory: A systematic review of human studies. Brain Sci..

[9] Cabrera Y., Koymans K.J., Poe G.R., Kessels H.W., Van Someren E.J.W., Wassing R. (2024). Overnight neuronal plasticity and adaptation to emotional distress. Nat. Rev. Neurosci..

[10] Battaglia S., Avenanti A., Vécsei L., Tanaka M. (2024). Neural Correlates and Molecular Mechanisms of Memory and Learning. Int. J. Mol. Sci..

[11] Lawal O., Ulloa Severino F.P., Eroglu C. (2022). The role of astrocyte structural plasticity in regulating neural circuit function and behavior. Glia.

[12] Herzberg M.P., Gunnar M.R. (2020). Early life stress and brain function: Activity and connectivity associated with processing emotion and reward. Neuroimage.

[13] Battaglia S., Di Fazio C., Mazzà M., Tamietto M., Avenanti A. (2024). Targeting Human Glucocorticoid Receptors in Fear Learning: A Multiscale Integrated Approach to Study Functional Connectivity. Int. J. Mol. Sci..

[14] Di Gregorio F., Steinhauser M., Maier M.E., Thayer J.F., Battaglia S. (2024). Error-related cardiac deceleration: Functional interplay between error-related brain activity and autonomic nervous system in performance monitoring. Neurosci. Biobehav. Rev..

[15] Battaglia S., Nazzi C., Thayer J.F. (2024). Genetic differences associated with dopamine and serotonin release mediate fear-induced bradycardia in the human brain. Transl. Psychiatry.

[16] Tanaka M., Chen C. (2023). Towards a mechanistic understanding of depression, anxiety, and their comorbidity: Perspectives from cognitive neuroscience. Front. Behav. Neurosci..

[17] Battaglia S., Schmidt A., Hassel S., Tanaka M. (2023). Case reports in neuroimaging and stimulation. Front. Psychiatry.

[18] Báez-Mendoza R., Vázquez Y., Mastrobattista E.P., Williams Z.M. (2021). Neuronal Circuits for Social Decision-Making and Their Clinical Implications. Front. Neurosci..

[19] Di Gregorio F., Battaglia S. (2023). Advances in EEG-based functional connectivity approaches to the study of the central nervous system in health and disease. Adv. Clin. Exp. Med..

[20] Duerler P., Vollenweider F.X., Preller K.H. (2022). A neurobiological perspective on social influence: Serotonin and social adaptation. J. Neurochem..

[21] Battaglia S., Nazzi C., Thayer J. (2023). Heart’s tale of trauma: Fear-conditioned heart rate changes in post-traumatic stress disorder. Acta Psychiatr. Scand..

[22] Valotto Neto L.J., Reverete de Araujo M., Moretti Junior R.C., Mendes Machado N., Joshi R.K., dos Santos Buglio D., Barbalho Lamas C., Direito R., Fornari Laurindo L., Tanaka M. (2024). Investigating the Neuroprotective and Cognitive-Enhancing Effects of Bacopa monnieri: A Systematic Review Focused on Inflammation, Oxidative Stress, Mitochondrial Dysfunction, and Apoptosis. Antioxidants.

[23] Martos D., Lőrinczi B., Szatmári I., Vécsei L., Tanaka M. (2024). The Impact of C-3 Side Chain Modifications on Kynurenic Acid: A Behavioral Analysis of Its Analogs on Motor Domain. Int. J. Mol. Sci..

[24] Jászberényi M., Thurzó B., Bagosi Z., Vécsei L., Tanaka M. (2024). The Orexin/Hypocretin System, the Peptidergic Regulator of Vigilance, Orchestrates Adaptation to Stress. Biomedicines.

[25] Tanaka M., Szabó Á., Körtési T., Szok D., Tajti J., Vécsei L. (2023). From CGRP to PACAP, VIP, and Beyond: Unraveling the Next Chapters in Migraine Treatment. Cells.

[26] Tajti J., Szok D., Csáti A., Szabó Á., Tanaka M., Vécsei L. (2023). Exploring novel therapeutic targets in the common pathogenic factors in migraine and neuropathic pain. Int. J. Mol. Sci..

[27] Bássoli R., Audi D., Ramalho B., Audi M., Quesada K., Barbalho S. (2023). The Effects of Curcumin on Neurodegenerative Diseases: A Systematic Review. J. Herb. Med..

[28] Buglio D.S., Marton L.T., Laurindo L.F., Guiguer E.L., Araújo A.C., Buchaim R.L., Goulart R.d.A., Rubira C.J., Barbalho S.M. (2022). The role of resveratrol in mild cognitive impairment and Alzheimer’s disease: A systematic review. J. Med. Food.

[29] Fraile-Ramos J., Garrit A., Reig-Vilallonga J., Giménez-Llort L. (2023). Hepatic Oxi-Inflammation and Neophobia as Potential Liver–Brain Axis Targets for Alzheimer’s Disease and Aging, with Strong Sensitivity to Sex, Isolation, and Obesity. Cells.

[30] Chen J., Huang L., Yang Y., Xu W., Qin Q., Qin R., Liang X., Lai X., Huang X., Xie M. (2023). Somatic Cell Reprogramming for Nervous System Diseases: Techniques, Mechanisms, Potential Applications, and Challenges. Brain Sci..

[31] Skobeleva K., Shalygin A., Mikhaylova E., Guzhova I., Ryazantseva M., Kaznacheyeva E. (2022). The STIM1/2-regulated calcium homeostasis is impaired in hippocampal neurons of the 5xFAD mouse model of Alzheimer’s disease. Int. J. Mol. Sci..

[32] Hong F., He G., Zhang M., Yu B., Chai C. (2022). The establishment of a mouse model of recurrent primary dysmenorrhea. Int. J. Mol. Sci..

[33] Garifulin R., Davleeva M., Izmailov A., Fadeev F., Markosyan V., Shevchenko R., Minyazeva I., Minekayev T., Lavrov I., Islamov R. (2023). Evaluation of the autologous genetically enriched leucoconcentrate on the lumbar spinal cord morpho-functional recovery in a mini pig with thoracic spine contusion injury. Biomedicines.

[34] Bueno C.R.d.S., Tonin M.C.C., Buchaim D.V., Barraviera B., Junior R.S.F., Santos P.S.d.S., Reis C.H.B., Pastori C.M., Pereira E.d.S.B.M., Nogueira D.M.B. (2023). Morphofunctional improvement of the facial nerve and muscles with repair using heterologous fibrin biopolymer and photobiomodulation. Pharmaceuticals.

[35] Kalkman H.O. (2023). Inhibition of microglial GSK3β activity is common to different kinds of antidepressants: A proposal for an in vitro screen to detect novel antidepressant principles. Biomedicines.

[36] Zheng Y., Huo J., Yang M., Zhang G., Wan S., Chen X., Zhang B., Liu H. (2022). ERK1/2 Signalling Pathway Regulates Tubulin-Binding Cofactor B Expression and Affects Astrocyte Process Formation after Acute Foetal Alcohol Exposure. Brain Sci..

[37] Li T., Xu G., Yi J., Huang Y. (2022). Intraoperative Hypothermia Induces Vascular Dysfunction in the CA1 Region of Rat Hippocampus. Brain Sci..

[38] Martos D., Tuka B., Tanaka M., Vécsei L., Telegdy G. (2022). Memory enhancement with kynurenic acid and its mechanisms in neurotransmission. Biomedicines.

[39] Sivananthan S., Lee L., Anderson G., Csanyi B., Williams R., Gissen P. (2023). Buffy coat score as a biomarker of treatment response in neuronal ceroid lipofuscinosis type 2. Brain Sci..

[40] Clement A., Wiborg O., Asuni A.A. (2020). Steps towards developing effective treatments for neuropsychiatric disturbances in Alzheimer’s disease: Insights from preclinical models, clinical data, and future directions. Front. Aging Neurosci..

[41] Socała K., Żmudzka E., Lustyk K., Zagaja M., Brighenti V., Costa A.M., Andres-Mach M., Pytka K., Martinelli I., Mandrioli J. (2024). Therapeutic potential of stilbenes in neuropsychiatric and neurological disorders: A comprehensive review of preclinical and clinical evidence. Phytother. Res..

[42] Statsenko Y., Habuza T., Smetanina D., Simiyu G.L., Meribout S., King F.C., Gelovani J.G., Das K.M., Gorkom K.N.-V., Zaręba K. (2023). Unraveling lifelong brain morphometric dynamics: A protocol for systematic review and meta-analysis in healthy neurodevelopment and ageing. Biomedicines.

[43] Fan P., Miranda O., Qi X., Kofler J., Sweet R.A., Wang L. (2023). Unveiling the enigma: Exploring risk factors and mechanisms for psychotic symptoms in Alzheimer’s disease through electronic medical records with deep learning models. Pharmaceuticals.

[44] Di Gregorio F., La Porta F., Petrone V., Battaglia S., Orlandi S., Ippolito G., Romei V., Piperno R., Lullini G. (2022). Accuracy of EEG biomarkers in the detection of clinical outcome in disorders of consciousness after severe acquired brain injury: Preliminary results of a pilot study using a machine learning approach. Biomedicines.

[45] Nani A., Manuello J., Mancuso L., Liloia D., Costa T., Vercelli A., Duca S., Cauda F. (2021). The pathoconnectivity network analysis of the insular cortex: A morphometric fingerprinting. NeuroImage.

[46] Cauda F., Nani A., Liloia D., Manuello J., Premi E., Duca S., Fox P.T., Costa T. (2020). Finding specificity in structural brain alterations through Bayesian reverse inference. Hum. Brain Mapp..

[47] Liloia D., Cauda F., Uddin L.Q., Manuello J., Mancuso L., Keller R., Nani A., Costa T. (2023). Revealing the selectivity of neuroanatomical alteration in autism spectrum disorder via reverse inference. Biol. Psychiatry Cogn. Neurosci. Neuroimaging.

[48] Liloia D., Crocetta A., Cauda F., Duca S., Costa T., Manuello J. (2022). Seeking overlapping neuroanatomical alterations between dyslexia and attention-deficit/hyperactivity disorder: A meta-analytic replication study. Brain Sci..

[49] Ippolito G., Bertaccini R., Tarasi L., Di Gregorio F., Trajkovic J., Battaglia S., Romei V. (2022). The role of alpha oscillations among the main neuropsychiatric disorders in the adult and developing human brain: Evidence from the last 10 years of research. Biomedicines.

[50] Zhao L., Hou B., Ji L., Ren D., Yuan F., Liu L., Bi Y., Yang F., Yu S., Yi Z. (2022). NGFR gene and single nucleotide polymorphisms, rs2072446 and rs11466162, playing roles in psychiatric disorders. Brain Sci..

[51] Khan S.R., Al Rijjal D., Piro A., Wheeler M.B. (2021). Integration of AI and traditional medicine in drug discovery. Drug Discov. Today.

[52] Barbalho S.M., Direito R., Laurindo L.F., Marton L.T., Guiguer E.L., Goulart R.d.A., Tofano R.J., Carvalho A.C., Flato U.A.P., Capelluppi Tofano V.A. (2022). Ginkgo biloba in the aging process: A narrative review. Antioxidants.

[53] Senevirathne D.K.L., Mahboob A., Zhai K., Paul P., Kammen A., Lee D.J., Yousef M.S., Chaari A. (2023). Deep brain stimulation beyond the clinic: Navigating the future of Parkinson’s and Alzheimer’s disease therapy. Cells.

[54] Vasiliu O. (2023). Efficacy, tolerability, and safety of toludesvenlafaxine for the treatment of major depressive disorder—A narrative review. Pharmaceuticals.

[55] Chu P.-C., Huang C.-S., Chang P.-K., Chen R.-S., Chen K.-T., Hsieh T.-H., Liu H.-L. (2023). Weak ultrasound contributes to neuromodulatory effects in the rat motor cortex. Int. J. Mol. Sci..

[56] Chojnowski K., Opiełka M., Gozdalski J., Radziwon J., Dańczyszyn A., Aitken A.V., Biancardi V.C., Winklewski P.J. (2023). The role of arginine-vasopressin in stroke and the potential use of arginine-vasopressin type 1 receptor antagonists in stroke therapy: A narrative review. Int. J. Mol. Sci..

[57] Adeel M., Chen C.-C., Lin B.-S., Chen H.-C., Liou J.-C., Li Y.-T., Peng C.-W. (2022). Safety of Special Waveform of Transcranial Electrical Stimulation (TES): In Vivo Assessment. Int. J. Mol. Sci..

[58] Tanaka M., Vécsei L. (2024). From Lab to Life: Exploring Cutting-Edge Models for Neurological and Psychiatric Disorders. Biomedicines.

[59] Tanaka M., Szabó Á., Vécsei L., Giménez-Llort L. (2023). Emerging translational research in neurological and psychiatric diseases: From in vitro to in vivo models. Int. J. Mol. Sci..

[60] Tanaka M., Szabó Á., Vécsei L. (2023). Preclinical modeling in depression and anxiety: Current challenges and future research directions. Adv. Clin. Exp. Med..

[61] Cabral D.F., Fried P., Koch S., Rice J., Rundek T., Pascual-Leone A., Sacco R., Wright C.B., Gomes-Osman J. (2022). Efficacy of mechanisms of neuroplasticity after a stroke. Restor. Neurol. Neurosci..

[62] Aderinto N., AbdulBasit M.O., Olatunji G., Adejumo T. (2023). Exploring the transformative influence of neuroplasticity on stroke rehabilitation: A narrative review of current evidence. Ann. Med. Surg..

[63] Motolese F., Capone F., Di Lazzaro V. (2022). New tools for shaping plasticity to enhance recovery after stroke. Handb. Clin. Neurol..

[64] Lim J.-S., Lee J.-J., Woo C.-W. (2021). Post-stroke cognitive impairment: Pathophysiological insights into brain disconnectome from advanced neuroimaging analysis techniques. J. Stroke.

[65] Griffis J.C., Metcalf N.V., Corbetta M., Shulman G.L. (2020). Damage to the shortest structural paths between brain regions is associated with disruptions of resting-state functional connectivity after stroke. NeuroImage.

[66] Rost N.S., Brodtmann A., Pase M.P., van Veluw S.J., Biffi A., Duering M., Hinman J.D., Dichgans M. (2022). Post-Stroke Cognitive Impairment and Dementia. Circ. Res..

[67] Zotey V., Andhale A., Shegekar T., Juganavar A. (2023). Adaptive Neuroplasticity in Brain Injury Recovery: Strategies and Insights. Cureus.

[68] De Luca C., Virtuoso A., Maggio N., Izzo S., Papa M., Colangelo A.M. (2020). Roadmap for Stroke: Challenging the Role of the Neuronal Extracellular Matrix. Int. J. Mol. Sci..

[69] Saceleanu V.M., Toader C., Ples H., Covache-Busuioc R.A., Costin H.P., Bratu B.G., Dumitrascu D.I., Bordeianu A., Corlatescu A.D., Ciurea A.V. (2023). Integrative Approaches in Acute Ischemic Stroke: From Symptom Recognition to Future Innovations. Biomedicines.

[70] d’Annunzio A., Arboix A., García-Eroles L., Sánchez-López M.-J. (2022). Vertigo in acute stroke is a predictor of brain location but is not related to early outcome: The experience of Sagrat Cor Hospital of Barcelona Stroke Registry. Biomedicines.

[71] Shimmyo K., Obayashi S. (2024). Fronto–Cerebellar Diaschisis and Cognitive Dysfunction after Pontine Stroke: A Case Series and Systematic Review. Biomedicines.

[72] Park S.Y., Lee S.P., Kim D., Kim W.J. (2023). Gut Dysbiosis: A New Avenue for Stroke Prevention and Therapeutics. Biomedicines.

[73] Li J., Li C., Subedi P., Tian X., Lu X., Miriyala S., Panchatcharam M., Sun H. (2023). Light Alcohol Consumption Promotes Early Neurogenesis Following Ischemic Stroke in Adult C57BL/6J Mice. Biomedicines.

[74] Gangemi A., De Luca R., Fabio R.A., Lauria P., Rifici C., Pollicino P., Marra A., Olivo A., Quartarone A., Calabrò R.S. (2023). Effects of Virtual Reality Cognitive Training on Neuroplasticity: A Quasi-Randomized Clinical Trial in Patients with Stroke. Biomedicines.

[75] Chen W.-C., Wang T.-S., Chang F.-Y., Chen P.-A., Chen Y.-C. (2023). Age, Dose, and Locomotion: Decoding Vulnerability to Ketamine in C57BL/6J and BALB/c Mice. Biomedicines.

[76] Nasini S., Tidei S., Shkodra A., De Gregorio D., Cambiaghi M., Comai S. (2023). Age-Related Effects of Exogenous Melatonin on Anxiety-like Behavior in C57/B6J Mice. Biomedicines.

[77] Fišar Z., Hroudová J., Zvěřová M., Jirák R., Raboch J., Kitzlerová E. (2023). Age-dependent alterations in platelet mitochondrial respiration. Biomedicines.

[78] Volnova A., Kurzina N., Belskaya A., Gromova A., Pelevin A., Ptukha M., Fesenko Z., Ignashchenkova A., Gainetdinov R.R. (2023). Noradrenergic modulation of learned and innate behaviors in dopamine transporter knockout rats by guanfacine. Biomedicines.

[79] Montanari M., Imbriani P., Bonsi P., Martella G., Peppe A. (2023). Beyond the microbiota: Understanding the role of the enteric nervous system in Parkinson’s disease from mice to human. Biomedicines.

[80] Chen B., Hasan M.M., Zhang H., Zhai Q., Waliullah A., Ping Y., Zhang C., Oyama S., Mimi M.A., Tomochika Y. (2023). UBL3 Interacts with Alpha-synuclein in Cells and the Interaction is Downregulated by the EGFR Pathway Inhibitor Osimertinib. Biomedicines.

[81] Chiarini A., Gui L., Viviani C., Armato U., Dal Prà I. (2023). NLRP3 Inflammasome’s Activation in Acute and Chronic Brain Diseases—An Update on Pathogenetic Mechanisms and Therapeutic Perspectives with Respect to Other Inflammasomes. Biomedicines.

[82] Polyák H., Galla Z., Nánási N., Cseh E.K., Rajda C., Veres G., Spekker E., Szabó Á., Klivényi P., Tanaka M. (2023). The tryptophan-kynurenine metabolic system is suppressed in cuprizone-induced model of demyelination simulating progressive multiple sclerosis. Biomedicines.

[83] Scalise S., Zannino C., Lucchino V., Lo Conte M., Scaramuzzino L., Cifelli P., D’Andrea T., Martinello K., Fucile S., Palma E. (2022). Human iPSC modeling of genetic febrile seizure reveals aberrant molecular and physiological features underlying an impaired neuronal activity. Biomedicines.

[84] Younes R., Issa Y., Jdaa N., Chouaib B., Brugioti V., Challuau D., Raoul C., Scamps F., Cuisinier F., Hilaire C. (2023). The Secretome of Human Dental Pulp Stem Cells and Its Components GDF15 and HB-EGF Protect Amyotrophic Lateral Sclerosis Motoneurons against Death. Biomedicines.

[85] Leone G.E., Shields D.C., Haque A., Banik N.L. (2023). Rehabilitation: Neurogenic Bone Loss after Spinal Cord Injury. Biomedicines.

[86] Gamboa O.L., Chuan-Peng H., Salas C.E., Yuen K.S. (2022). Obliviate! Reviewing Neural Fundamentals of Intentional Forgetting from a Meta-Analytic Perspective. Biomedicines.

[87] Battaglia S., Fabius J.H., Moravkova K., Fracasso A., Borgomaneri S. (2022). The neurobiological correlates of gaze perception in healthy individuals and neurologic patients. Biomedicines.

[88] Yoshimura R., Okamoto N., Chibaatar E., Natsuyama T., Ikenouchi A. (2023). The serum brain-derived neurotrophic factor increases in serotonin reuptake inhibitor responders patients with first-episode, drug-naïve major depression. Biomedicines.

[89] Cremone I.M., Nardi B., Amatori G., Palego L., Baroni D., Casagrande D., Massimetti E., Betti L., Giannaccini G., Dell’Osso L. (2023). Unlocking the secrets: Exploring the biochemical correlates of suicidal thoughts and behaviors in adults with autism spectrum conditions. Biomedicines.

[90] Parolini F., Goethel M., Becker K., Fernandes C., Fernandes R.J., Ervilha U.F., Santos R., Vilas-Boas J.P. (2023). Breaking Barriers: Artificial Intelligence Interpreting the Interplay between Mental Illness and Pain as Defined by the International Association for the Study of Pain. Biomedicines.

[91] Man Chan Y., Wong Y., Khalid N., Wastling S., Flores-Martin A., Frank L.A., Koohi N., Arshad Q., Davagnanam I., Kaski D. (2021). Prevalence of acute dizziness and vertigo in cortical stroke. Eur. J. Neurol..

[92] Janacsek K., Evans T.M., Kiss M., Shah L., Blumenfeld H., Ullman M.T. (2022). Subcortical Cognition: The Fruit Below the Rind. Annu. Rev. Neurosci..

[93] Cervellati C., Trentini A., Pecorelli A., Valacchi G. (2020). Inflammation in Neurological Disorders: The Thin Boundary Between Brain and Periphery. Antioxid. Redox Signal.

[94] Chen Y., Xu J., Chen Y. (2021). Regulation of Neurotransmitters by the Gut Microbiota and Effects on Cognition in Neurological Disorders. Nutrients.

[95] Elkind M.S., Sciacca R., Boden-Albala B., Rundek T., Paik M.C., Sacco R.L. (2006). Moderate alcohol consumption reduces risk of ischemic stroke: The Northern Manhattan Study. Stroke.

[96] Shiotsuki H., Saijo Y., Ogushi Y., Kobayashi S. (2022). Relationship between Alcohol Intake and Stroke Severity in Japanese Patients: A Sex- and Subtype-Stratified Analysis. J. Stroke Cerebrovasc. Dis..

[97] Faria A.L., Pinho M.S., Bermúdez I.B.S. (2020). A comparison of two personalization and adaptive cognitive rehabilitation approaches: A randomized controlled trial with chronic stroke patients. J. Neuroeng. Rehabil..

[98] Xuefang L., Guihua W., Fengru M. (2021). The effect of early cognitive training and rehabilitation for patients with cognitive dysfunction in stroke. Int. J. Methods Psychiatr. Res..

[99] VanGilder J.L., Hooyman A., Peterson D.S., Schaefer S.Y. (2020). Post-stroke cognitive impairments and responsiveness to motor rehabilitation: A review. Curr. Phys. Med. Rehabil. Rep..

[100] van Balkom T.D., van den Heuvel O.A., Berendse H.W., van der Werf Y.D., Vriend C. (2020). The Effects of Cognitive Training on Brain Network Activity and Connectivity in Aging and Neurodegenerative Diseases: A Systematic Review. Neuropsychol. Rev..

[101] Stumme J., Jockwitz C., Hoffstaedter F., Amunts K., Caspers S. (2020). Functional network reorganization in older adults: Graph-theoretical analyses of age, cognition and sex. Neuroimage.

[102] Anatürk M., Kaufmann T., Cole J.H., Suri S., Griffanti L., Zsoldos E., Filippini N., Singh-Manoux A., Kivimäki M., Westlye L.T. (2021). Prediction of brain age and cognitive age: Quantifying brain and cognitive maintenance in aging. Hum. Brain Mapp..

[103] Walsh Z., Mollaahmetoglu O.M., Rootman J., Golsof S., Keeler J., Marsh B., Nutt D.J., Morgan C.J.A. (2021). Ketamine for the treatment of mental health and substance use disorders: Comprehensive systematic review. BJPsych Open.

[104] Hartelius G., Muscat S.A., Bartova L. (2023). Editorial: Bridging the gap: An interdisciplinary perspective on ketamine in psychiatric disorders. Front. Psychiatry.

[105] Corkery J.M., Hung W.C., Claridge H., Goodair C., Copeland C.S., Schifano F. (2021). Recreational ketamine-related deaths notified to the National Programme on Substance Abuse Deaths, England, 1997–2019. J. Psychopharmacol..

[106] Biggio G., Biggio F., Talani G., Mostallino M.C., Aguglia A., Aguglia E., Palagini L. (2021). Melatonin: From Neurobiology to Treatment. Brain Sci..

[107] Anghel L., Baroiu L., Popazu C.R., Pătraș D., Fotea S., Nechifor A., Ciubara A., Nechita L., Mușat C.L., Stefanopol I.A. (2022). Benefits and adverse events of melatonin use in the elderly (Review). Exp. Ther. Med..

[108] Gunata M., Parlakpinar H., Acet H.A. (2020). Melatonin: A review of its potential functions and effects on neurological diseases. Rev. Neurol..

[109] de Oliveira Zanuso B., Dos Santos A.R.d.O., Miola V.F.B., Campos L.M.G., Spilla C.S.G., Barbalho S.M. (2022). Panax ginseng and aging related disorders: A systematic review. Exp. Gerontol..

[110] de Souza G.A., de Marqui S.V., Matias J.N., Guiguer E.L., Barbalho S.M. (2020). Effects of Ginkgo biloba on diseases related to oxidative stress. Planta Medica.

[111] Tripp G., Wickens J. (2024). Using rodent data to elucidate dopaminergic mechanisms of ADHD: Implications for human personality. Personal. Neurosci..

[112] Kanarik M., Grimm O., Mota N.R., Reif A., Harro J. (2022). ADHD co-morbidities: A review of implication of gene × environment effects with dopamine-related genes. Neurosci. Biobehav. Rev..

[113] Cannon Homaei S., Barone H., Kleppe R., Betari N., Reif A., Haavik J. (2022). ADHD symptoms in neurometabolic diseases: Underlying mechanisms and clinical implications. Neurosci. Biobehav. Rev..

[114] Feigin V.L., Vos T., Nichols E., Owolabi M.O., Carroll W.M., Dichgans M., Deuschl G., Parmar P., Brainin M., Murray C. (2020). The global burden of neurological disorders: Translating evidence into policy. Lancet Neurol..

[115] Dumurgier J., Tzourio C. (2020). Epidemiology of neurological diseases in older adults. Rev. Neurol..

[116] Popa-Wagner A., Dumitrascu D.I., Capitanescu B., Petcu E.B., Surugiu R., Fang W.H., Dumbrava D.A. (2020). Dietary habits, lifestyle factors and neurodegenerative diseases. Neural Regen. Res..

[117] Mey G.M., Mahajan K.R., DeSilva T.M. (2023). Neurodegeneration in multiple sclerosis. WIREs Mech. Dis..

[118] Ratan Y., Rajput A., Maleysm S., Pareek A., Jain V., Pareek A., Kaur R., Singh G. (2023). An Insight into Cellular and Molecular Mechanisms Underlying the Pathogenesis of Neurodegeneration in Alzheimer’s Disease. Biomedicines.

[119] Mishra A.K., Dixit A. (2022). Dopaminergic Axons: Key Recitalists in Parkinson’s Disease. Neurochem. Res..

[120] Masrori P., Van Damme P. (2020). Amyotrophic lateral sclerosis: A clinical review. Eur. J. Neurol..

[121] Direito R., Barbalho S.M., Sepodes B., Figueira M.E. (2024). Plant-Derived Bioactive Compounds: Exploring Neuroprotective, Metabolic, and Hepatoprotective Effects for Health Promotion and Disease Prevention. Pharmaceutics.

[122] Natale G., Ryskalin L., Morucci G., Lazzeri G., Frati A., Fornai F. (2021). The Baseline Structure of the Enteric Nervous System and Its Role in Parkinson’s Disease. Life.

[123] Niesler B., Kuerten S., Demir I.E., Schäfer K.H. (2021). Disorders of the enteric nervous system—A holistic view. Nat. Rev. Gastroenterol. Hepatol..

[124] Chanpong A., Borrelli O., Thapar N. (2022). Recent advances in understanding the roles of the enteric nervous system. Fac. Rev..

[125] Hwang J.T., Lee A., Kho C. (2022). Ubiquitin and Ubiquitin-like Proteins in Cancer, Neurodegenerative Disorders, and Heart Diseases. Int. J. Mol. Sci..

[126] He S., Wang F., Yung K.K.L., Zhang S., Qu S. (2021). Effects of α-Synuclein-Associated Post-Translational Modifications in Parkinson’s Disease. ACS Chem. Neurosci..

[127] Sahoo S., Padhy A.A., Kumari V., Mishra P. (2022). Role of Ubiquitin-Proteasome and Autophagy-Lysosome Pathways in α-Synuclein Aggregate Clearance. Mol. Neurobiol..

[128] Fornari Laurindo L., Aparecido Dias J., Cressoni Araújo A., Torres Pomini K., Machado Galhardi C., Rucco Penteado Detregiachi C., Santos de Argollo Haber L., Donizeti Roque D., Dib Bechara M., Vialogo Marques de Castro M. (2024). Immunological dimensions of neuroinflammation and microglial activation: Exploring innovative immunomodulatory approaches to mitigate neuroinflammatory progression. Front. Immunol..

[129] Spiteri A.G., Wishart C.L., Pamphlett R., Locatelli G., King N.J.C. (2022). Microglia and monocytes in inflammatory CNS disease: Integrating phenotype and function. Acta Neuropathol..

[130] Piancone F., La Rosa F., Marventano I., Saresella M., Clerici M. (2021). The Role of the Inflammasome in Neurodegenerative Diseases. Molecules.

[131] Fathi M., Vakili K., Yaghoobpoor S., Tavasol A., Jazi K., Mohamadkhani A., Klegeris A., McElhinney A., Mafi Z., Hajiesmaeili M. (2022). Dynamic changes in kynurenine pathway metabolites in multiple sclerosis: A systematic review. Front. Immunol..

[132] Isık S.M.T., Onmaz D.E., Ekmekci A.H., Ozturk S., Unlu A., Abusoglu S. (2023). Relationship of tryptophan metabolites with the type and severity of multiple sclerosis. Mult. Scler. Relat. Disord..

[133] Török N., Tanaka M., Vécsei L. (2020). Searching for peripheral biomarkers in neurodegenerative diseases: The tryptophan-kynurenine metabolic pathway. Int. J. Mol. Sci..

[134] Ohno Y., Ishihara S., Mashimo T., Sofue N., Shimizu S., Imaoku T., Tsurumi T., Sasa M., Serikawa T. (2011). Scn1a missense mutation causes limbic hyperexcitability and vulnerability to experimental febrile seizures. Neurobiol. Dis..

[135] Dutton S.B., Dutt K., Papale L.A., Helmers S., Goldin A.L., Escayg A. (2017). Early-life febrile seizures worsen adult phenotypes in Scn1a mutants. Exp. Neurol..

[136] Yi Y., Zhong C., Wei-Wei H. (2023). The long-term neurodevelopmental outcomes of febrile seizures and underlying mechanisms. Front. Cell Dev. Biol..

[137] Gugliandolo A., Mazzon E. (2021). Dental mesenchymal stem cell secretome: An intriguing approach for neuroprotection and neuroregeneration. Int. J. Mol. Sci..

[138] Ueda T., Inden M., Ito T., Kurita H., Hozumi I. (2020). Characteristics and therapeutic potential of dental pulp stem cells on neurodegenerative diseases. Front. Neurosci..

[139] Santilli F., Fabrizi J., Santacroce C., Caissutti D., Spinello Z., Candelise N., Lancia L., Pulcini F., Delle Monache S., Mattei V. (2024). Analogies and Differences between Dental Stem Cells: Focus on Secretome in Combination with Scaffolds in Neurological Disorders. Stem Cell Rev. Rep..

[140] Invernizzi M., De Sire A., Renò F., Cisari C., Runza L., Baricich A., Carda S., Fusco N. (2020). Spinal cord injury as a model of bone-muscle interactions: Therapeutic implications from in vitro and in vivo studies. Front. Endocrinol..

[141] Invernizzi M., de Sire A., Carda S., Venetis K., Renò F., Cisari C., Fusco N. (2020). Bone muscle crosstalk in spinal cord injuries: Pathophysiology and implications for patients’ quality of life. Curr. Osteoporos. Rep..

[142] Otzel D.M., Kok H.J., Graham Z.A., Barton E.R., Yarrow J.F. (2021). Pharmacologic approaches to prevent skeletal muscle atrophy after spinal cord injury. Curr. Opin. Pharmacol..

[143] Cerna C., García F.E., Téllez A. (2020). Brief mindfulness, mental health, and cognitive processes: A randomized controlled trial. PsyCh J..

[144] Denecke K., Vaaheesan S., Arulnathan A. (2020). A mental health chatbot for regulating emotions (SERMO)-concept and usability test. IEEE Trans. Emerg. Top. Comput..

[145] Pachucki M.C., Ozer E.J., Barrat A., Cattuto C. (2015). Mental health and social networks in early adolescence: A dynamic study of objectively-measured social interaction behaviors. Soc. Sci. Med..

[146] Matias J.N., Achete G., Campanari G.S.d.S., Guiguer É.L., Araújo A.C., Buglio D.S., Barbalho S.M. (2021). A systematic review of the antidepressant effects of curcumin: Beyond monoamines theory. Aust. N. Z. J. Psychiatry.

[147] Costanzi M., Cianfanelli B., Santirocchi A., Lasaponara S., Spataro P., Rossi-Arnaud C., Cestari V. (2021). Forgetting Unwanted Memories: Active Forgetting and Implications for the Development of Psychological Disorders. J. Pers. Med..

[148] Anderson M.C., Hulbert J.C. (2021). Active Forgetting: Adaptation of Memory by Prefrontal Control. Annu. Rev. Psychol..

[149] Luo Y., Wang R., Xie H., He Z. (2024). The interplay between memory control and emotion regulation. Ann. N. Y. Acad. Sci..

[150] McKay K.T., Grainger S.A., Coundouris S.P., Skorich D.P., Phillips L.H., Henry J.D. (2021). Visual attentional orienting by eye gaze: A meta-analytic review of the gaze-cueing effect. Psychol. Bull..

[151] Kompatsiari K., Ciardo F., Wykowska A. (2022). To follow or not to follow your gaze: The interplay between strategic control and the eye contact effect on gaze-induced attention orienting. J. Exp. Psychol. Gen..

[152] Hadders-Algra M. (2022). Human face and gaze perception is highly context specific and involves bottom-up and top-down neural processing. Neurosci. Biobehav. Rev..

[153] Domínguez-Borràs J., Vuilleumier P. (2022). Amygdala function in emotion, cognition, and behavior. Handb. Clin. Neurol..

[154] Barton J.J.S. (2022). Face processing in the temporal lobe. Handb. Clin. Neurol..

[155] Rana T., Behl T., Sehgal A., Srivastava P., Bungau S. (2021). Unfolding the Role of BDNF as a Biomarker for Treatment of Depression. J. Mol. Neurosci..

[156] Zelada M.I., Garrido V., Liberona A., Jones N., Zúñiga K., Silva H., Nieto R.R. (2023). Brain-Derived Neurotrophic Factor (BDNF) as a Predictor of Treatment Response in Major Depressive Disorder (MDD): A Systematic Review. Int. J. Mol. Sci..

[157] Nikolac Perkovic M., Gredicak M., Sagud M., Nedic Erjavec G., Uzun S., Pivac N. (2023). The association of brain-derived neurotrophic factor with the diagnosis and treatment response in depression. Expert. Rev. Mol. Diagn..

[158] Abou Chahla M.N., Khalil M.I., Comai S., Brundin L., Erhardt S., Guillemin G.J. (2023). Biological Factors Underpinning Suicidal Behaviour: An Update. Brain Sci..

[159] Berkelmans G., van der Mei R., Bhulai S., Gilissen R. (2021). Identifying socio-demographic risk factors for suicide using data on an individual level. BMC Public Health.

[160] Roy B., Ochi S., Dwivedi Y. (2023). Potential of Circulating miRNAs as Molecular Markers in Mood Disorders and Associated Suicidal Behavior. Int. J. Mol. Sci..

[161] Lang V.A., Lundh T., Ortiz-Catalan M. (2021). Mathematical and Computational Models for Pain: A Systematic Review. Pain. Med..

[162] Markfelder T., Pauli P. (2020). Fear of pain and pain intensity: Meta-analysis and systematic review. Psychol. Bull..

[163] Puschmann A.K., Drießlein D., Beck H., Arampatzis A., Moreno Catalá M., Schiltenwolf M., Mayer F., Wippert P.M. (2020). Stress and Self-Efficacy as Long-Term Predictors for Chronic Low Back Pain: A Prospective Longitudinal Study. J. Pain. Res..

[164] Tanaka M., Diano M., Battaglia S. (2023). Insights into structural and functional organization of the brain: Evidence from neuroimaging and non-invasive brain stimulation techniques. Front. Psychiatry.

[165] Candini M., Battaglia S., Benassi M., di Pellegrino G., Frassinetti F. (2021). The physiological correlates of interpersonal space. Sci. Rep..

[166] Ellena G., Battaglia S., Làdavas E. (2020). The spatial effect of fearful faces in the autonomic response. Exp. Brain Res..

[167] Mendes A.J., de Souza Greco A.I., Pereira R.S., Malfará W.R., de Souza M.d.S.S., Barbalho S.M., Guiguer E.L., Araujo A.C. (2022). Evaluation of the anxiolytic effects of acute administration of Passiflora alata extract in wistar rats submitted to swimming. J. Med. Plants Res..

